# Nanostructured lipid carriers for percutaneous administration of alkaloids isolated from *Aconitum sinomontanum*

**DOI:** 10.1186/s12951-015-0107-3

**Published:** 2015-07-10

**Authors:** Teng Guo, Yongtai Zhang, Jihui Zhao, Chunyun Zhu, Nianping Feng

**Affiliations:** Department of Pharmaceutical Sciences, School of Pharmacy, Shanghai University of Traditional Chinese Medicine, 1200 Cailun Road, Shanghai, 201203 People’s Republic of China

**Keywords:** Nanostructured lipid carriers, Solid lipid nanoparticles, Transdermal delivery, Cellular uptake, Microdialysis

## Abstract

**Background:**

Lipid-based nanosystems have great potential for transdermal drug delivery. In this study, nanostructured lipid carriers (NLCs) for short-acting alkaloids lappacontine (LA) and ranaconitine (RAN) isolated from *Aconitum sinomontanum* (AAS) at 69.47 and 9.16% (w/w) yields, respectively, were prepared to enhance percutaneous permeation. Optimized NLC formulations were evaluated using uniform design experiments. Microstructure and in vitro/in vivo transdermal delivery characteristics of AAS-loaded NLCs and solid lipid nanoparticles (SLNs) were compared. Cellular uptake of fluorescence-labeled nanoparticles was probed using laser scanning confocal microscopy and fluorescence-activated cell sorting. Nanoparticle integrity during transdermal delivery and effects on the skin surface were also investigated.

**Results:**

NLC formulations were less cytotoxic than the AAS solution in HaCaT and CCC-ESF cells. Moreover, coumarin-6-labeled NLCs showed biocompatibility with HaCaT and CCC-ESF cells, and their cellular uptake was strongly affected by cholesterol and lipid rafts. Significantly greater cumulative amounts of NLC-associated LA and RAN than SLN-associated alkaloids penetrated the rat skin in vitro. In vivo microdialysis showed higher area under the concentration–time curve (AUC)_0–t_ for AAS-NLC-associated LA and RAN than for AAS-SLN-associated alkaloids.

**Conclusions:**

NLC formulations could be good transdermal systems for increasing biocompatibility and decreasing cytotoxicity of AAS. AAS-NLCs showed higher percutaneous permeation than the other preparations. These findings suggest that NLCs could be promising transdermal delivery vehicles for AAS.

## Background

*Aconitum sinomontanum* (AS) belongs to the *Ranunculaceae* family and is used extensively as an analgesic and antirheumatic agent in traditional Chinese medicine [1]. The pharmacological effects of AS are attributed to diterpenoid and norditerpenoid alkaloids, primarily lappaconitine and ranaconitine [2]. Recently, total alkaloid extracts of *A. sinomontanum* (AAS) have been used as pain relievers for patients with cancer [3]. The major metabolic pathways of the main components of AAS in rat urine are hydroxylation, *O*-demethylation, *N*-deacetylation, and ester hydrolyzation [4]. However, the oral bioavailability of AAS is poor, and its main components including lappaconitine and ranaconitine have a relatively short half-life. Therefore, they require long-term oral administration. It was reported that lappaconitine has certain toxicities and its oral 50% lethal doses (LD_50_) in mice and rats are 32.4 and 20 mg/kg, respectively [5]. Besides, its certain safety factor (CSF) and standard safety margin (SSM) are 1.01 and 1.0% for per os, respectively, which indicates a narrow therapeutic range and low therapeutic index with oral administration [6]. Transdermal delivery is superior to oral administration as a route for AAS because it avoids the first-pass metabolism in the liver. In addition, this route reduces drug toxicity and adverse reactions by maintaining stable and lasting blood drug concentrations [7].

Lipid-based nanosystems are novel colloidal nanocarriers that are used for transdermal drug delivery [8]. Solid lipid nanoparticles (SLNs) and nanostructured lipid carriers (NLCs) are two types of lipid nanoparticles that have been shown to enhance transdermal permeation [9]. Compared with other transdermal vehicles such as creams, tinctures, emulsions, and liposomes lipid nanoparticles exhibit negligible skin irritation, higher stability, and more controlled release [10]. NLCs consist of a mixture of solid and liquid lipids and represent the second-generation of lipid nanoparticle-based drug vehicles [11]. NLCs have the beneficial properties of SLNs and overcome their limitations including limited drug-loading capacity, drug leakage during storage, and risk of gelation [12, 13]. Furthermore, NLCs have been confirmed to produce efficient transdermal drug permeation [14].

In the current study, a range of AAS-NLCs was successfully prepared to optimize the vehicle formulation. The microstructure and in vitro/in vivo transdermal delivery characteristics of AAS-loaded NLCs were investigated and compared with those of AAS-loaded SLNs. Laser scanning confocal microscopy (LSCM) and fluorescence-activated cell sorting (FACS) were used to probe the cellular uptake of fluorescence-labeled nanoparticles by human epidermal keratinocyte (HaCaT) and human embryonic skin fibroblast (CCC-ESF) cell lines. To elucidate the transdermal delivery mechanism of NLCs, the integrity of the nanoparticles during transdermal delivery and their effects on the skin surface were also investigated.

## Results and discussion

### Preparation of AAS-NLCs

**Figure 1:**
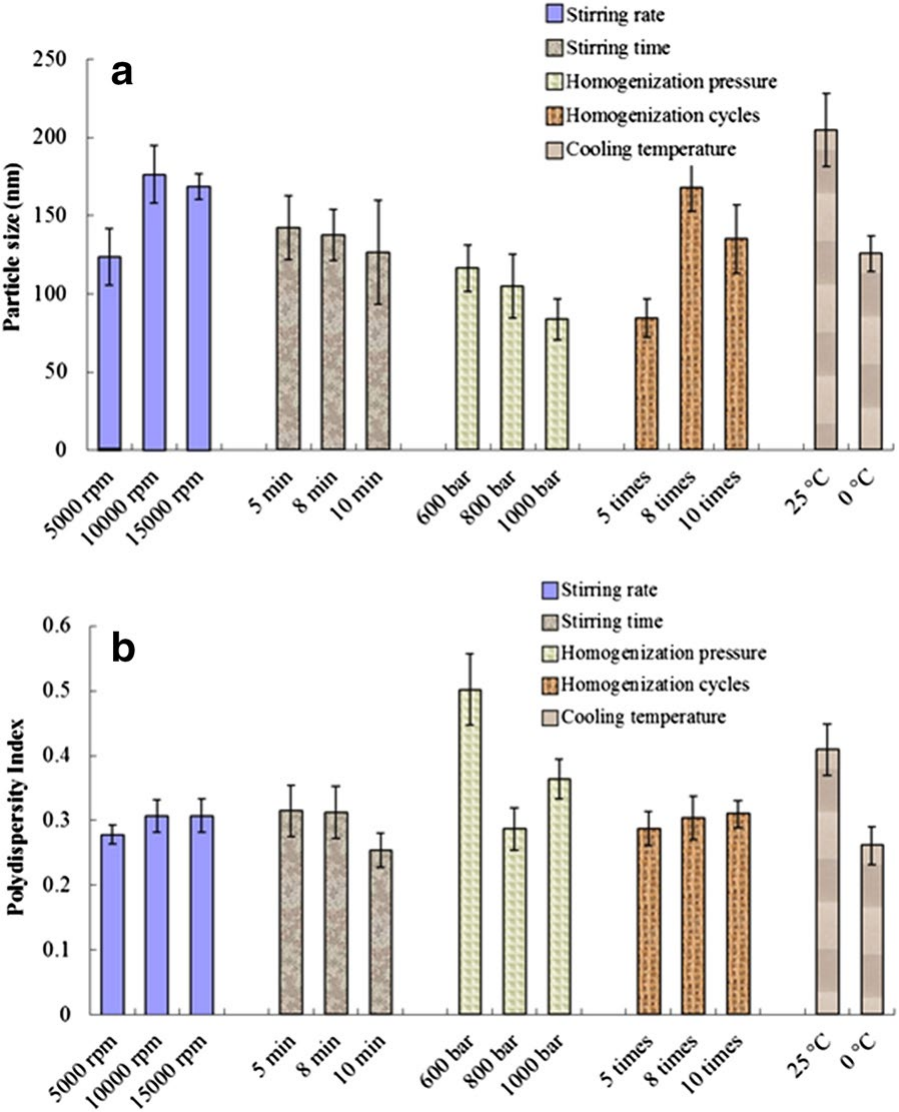
Effect of optimization of the process parameters of high-pressure homogenization method. On **a** particle size and **b** polydispersity index.

The stirring rate (rpm), stirring time (min), homogenization pressure (bar), homogenization cycle, and cooling temperature were evaluated to optimize the preparation of the AAS-NLCs. As shown in Figure 1, the particle size and polydispersity index (PDI) were reduced as the stirring rate was slowed or the stirring time was lengthened. As the homogenization pressure increased, the particle size and PDI were reduced but subsequently increased if the pressure continued to increase. Moreover, particle size decreased as the number of homogenization cycles decreased. In addition, a small particle size and low PDI were produced by cooling the formulation to room temperature in an ice bath. It was previously reported that the instability of the preparation system is caused by an increase in the kinetic energy of droplets due to the increase in homogenization pressure and number of homogenization cycles [15]. Therefore, the production parameters were set as follows: stirring rate, 5,000 rpm; stirring time, 10 min; homogenization pressure, 800 bar; cooling temperature, 0°C; and five homogenization cycles were performed.

The compositions of the NLC formulations are presented in Tables 1 and 2. A multiple linear regression equation was generated using a linear multiple stepwise regression analysis as follows: Y_1_ = −13.663X_2_X_3_ + 408.072 (R^2^ = 0.755, *p* < 0.05); Y_2_ = 53.214X_1_X_4_ – 2,278.216X_4_^2^ + 57.246 (R^2^ = 0.952, *p* < 0.01); Y_3_ = 2.812X_1_ + 45.071 (R^2^ = 0.890, *p* < 0.01); Y_4_ = 75.962X_4_ − 0.584 (R^2^ = 0.952, *p* < 0.01); Y_5_ = 0.83X_1_X_4_ − 2.019X_3_X_4_ + 0.023 (R^2^ = 0.996, *p* < 0.01). Most of the R^2^ values were higher than 0.9 and the *p* values were less than 0.05, indicating that the goodness of fitting was fine and the significant effects were generated from the factors on the responses, respectively. A multi-grid algorithm was used to identify suitable factors required to produce a particle size of less than 200 nm. These were, an entrapment efficiency (EE) for LA and RAN greater than 80 and 70%, respectively, and drug loading (DL) for LA and RAN greater than 4 and 0.2%, respectively. In the optimal formulation, the concentration of lipids, concentration of surfactants, solid/liquid ratio, and drug/lipid ratio were 9%, 5.5%, 2.91, and 0.1, respectively.

**Table 1:** Compositions of NLC formulations used in the optimization experiments [U*7(7^4^) uniform experimental design]

NLCs	Factor A (X_1_)	Factor B (X_2_)	Factor C (X_3_)	Factor D (X_4_)	Drug content
	Lipid (%)	Surfactant (%)	Solid/liquid (w/w)	Drug/lipid (w/w)	(%, w/v)
NLC1	2	4	1:1.5	1:30	0.07
NLC2	4	6	2:1	1:25	0.16
NLC3	6	8	1:2	1:20	0.30
NLC4	8	3	1.5:1	1:15	0.53
NLC5	10	5	1:3	1:10	1.00
NLC6	12	7	1:1	1:5	2.40
NLC7	14	9	3:1	1:35	0.40

**Table 2:** Results of NLC formulation optimization experiment using a U*7(7^4^) uniform experimental design (mean ± standard deviation, n = 3)

NLCs	Response (Y_1_)	Response (Y_2_)	Response (Y_3_)	Response (Y_4_)	Response (Y_5_)
	Size (nm)	EE (LA, %)	EE (RAN, %)	DL (LA, %)	DL (RAN, %)
NLC1	374.0 ± 25.7	54.70 ± 7.23	48.93 ± 5.63	0.78 ± 0.05	0.04 ± 0.01
NLC2	137.2 ± 11.3	66.82 ± 2.66	57.78 ± 1.97	1.33 ± 0.12	0.02 ± 0.01
NLC3	353.4 ± 28.5	66.07 ± 8.63	65.27 ± 9.73	2.14 ± 0.11	0.12 ± 0.02
NLC4	508.7 ± 68.7	81.45 ± 5.92	57.46 ± 3.24	6.67 ± 0.58	0.26 ± 0.02
NLC5	424.3 ± 41.2	88.25 ± 7.22	80.84 ± 8.45	8.89 ± 0.57	0.85 ± 0.02
NLC6	157.9 ± 20.3	93.14 ± 6.12	83.86 ± 7.23	13.58 ± 1.21	1.59 ± 0.09
NLC7	97.1 ± 11.2	71.31 ± 4.32	78.85 ± 6.11	1.91 ± 0.22	0.21 ± 0.02

### Characterizations of AAS-NLCs

The particle size and PDI are important characteristics of NLCs that influence the distribution of nanoparticles [16]. AAS-NLCs and AAS-SLNs had PDI values of 0.082 ± 0.009 and 0.073 ± 0.006, respectively and narrow size distributions (160.5 ± 7.9 and 176.6 ± 19.6 nm for AAS-NLCs and AAS-SLNs, respectively). The ZP values of the AAS-NLCs and AAS-SLNs were −24.8 ± 1.6 and −20.3 ± 2.1 mV, respectively, indicating good stability in these systems. In addition, the EE and DL of the NLC-associated LA were 82.18 ± 1.93 and 4.55 ± 0.52%, respectively while the EE and DL of NLC-associated RAN were 81.87 ± 1.52 and 0.36 ± 0.01%, respectively. The EE and DL of the SLN-associated LA were 77.48 ± 2.56 and 3.61 ± 0.75%, respectively, and the EE and DL of SLN-associated RAN were 74.81 ± 1.97 and 0.21 ± 0.01%, respectively.

**Figure 2:**
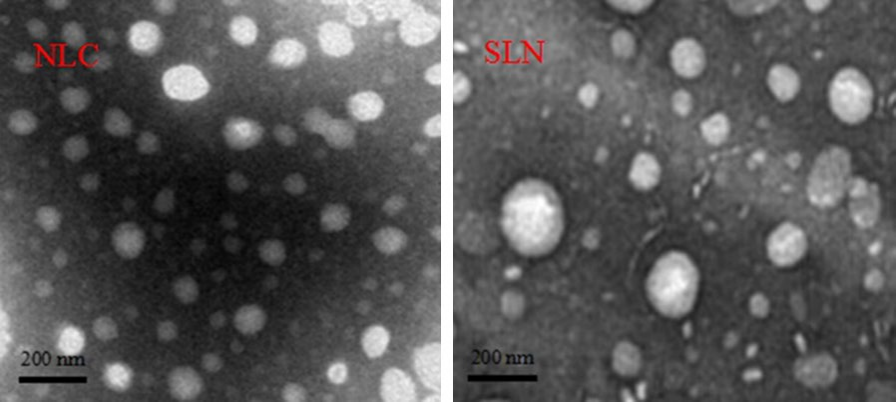
TEM images. Representative images of AAS-NLCs and AAS-SLNs (×80,000 magnification).

The morphologies of the AAS-NLCs and AAS-SLNs were observed using transmission electron microscopy (TEM). As shown in Figure 2, most of the particles were spheroidal and uniform in size.

**Figure 3:**
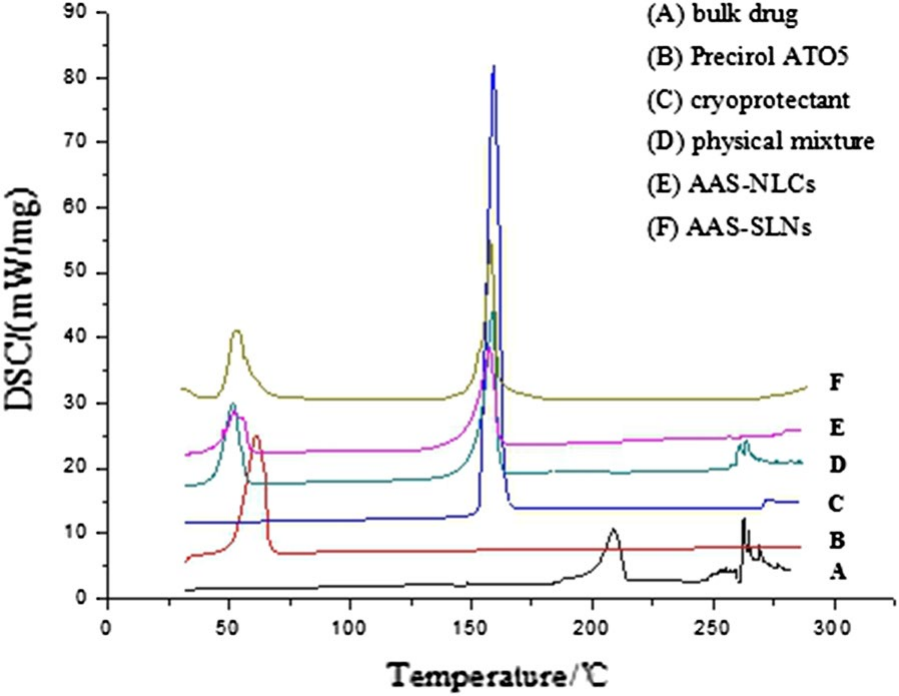
DSC curves. Representative DSC curves of *A* bulk drug, *B* Precirol ATO5, *C* cryoprotectant, *D* physical mixture, *E* AAS-NLCs, and *F* AAS-SLNs.

Differential scanning calorimetry (DSC) was performed to investigate the melting and crystallization behavior of the lipid molecules and drugs in the nanoparticle preparations [17]. As shown in Figure 3, the bulk drug had endothermic peaks at 208.6 and 269.0°C, the Precirol ATO 5 displayed a melting point of 61.1°C, and the cryoprotectant (mannitol) exhibited an endothermic peak at 159.3°C. The physical mixture heating curves exhibited three endothermic peaks that corresponded to the melting points of the three components. These included the reduced endothermic peak of the bulk drug at 269.0°C and the moving endothermic peak of Precirol ATO 5 at 51.5°C. The results indicated that the enhanced dissolution of the partial bulk drug in the solid lipids as the temperature rose led to changes in crystalline structure. All the endothermic peaks of AAS disappeared and the peak of Precirol ATO 5 was significantly shifted to the right in the AAS-NLC and AAS-SLN heating curves, indicating an increase in lattice defects in the AAS-NLC and AAS-SLN preparations. The results suggest that the drug was homogeneously dispersed in the nanoparticles in a disordered crystalline state.

**Figure 4:**
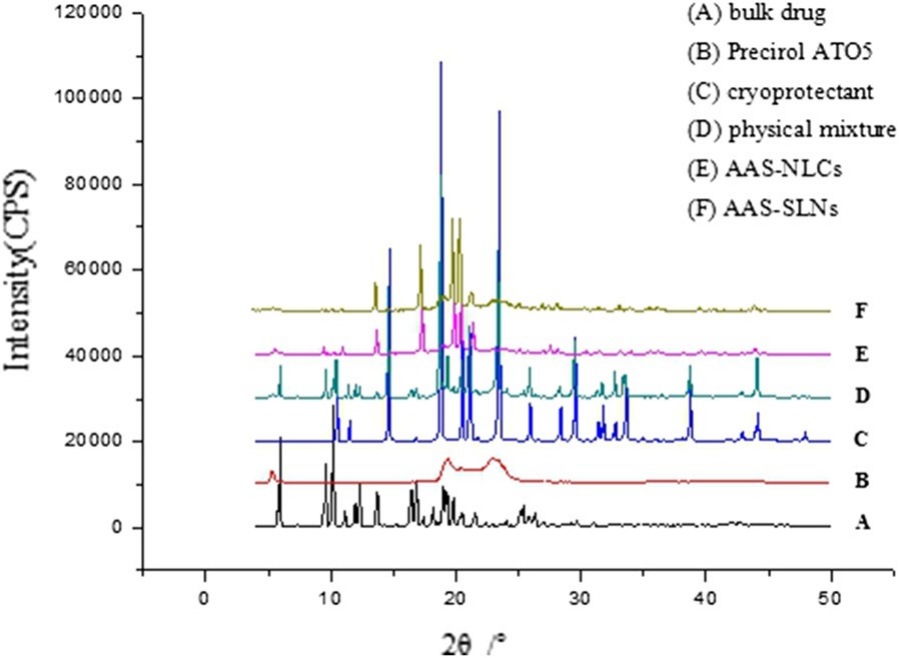
XRD spectra. Representative XRD of *A* bulk drug, *B* Precirol ATO5, *C* cryoprotectant, *D* physical mixture, *E* AAS-NLCs, and *F* AAS-SLNs.

X-ray diffraction (XRD) and DSC are complementary analytical methods used in the assessment of crystallinity and polymorphism [18]. Unlike the physical mixture (Figure 4D), the characteristic peaks of AAS (Figure 4A), precirol ATO 5 (Figure 4B), and mannitol (Figure 4C) in the XRD graph of the nanoparticles preparation (Figure 4E, F) were absent or reduced, confirming the formation of disordered structures in the inner cores of the NLC and SLN particles.

### In vitro release

**Figure 5:**
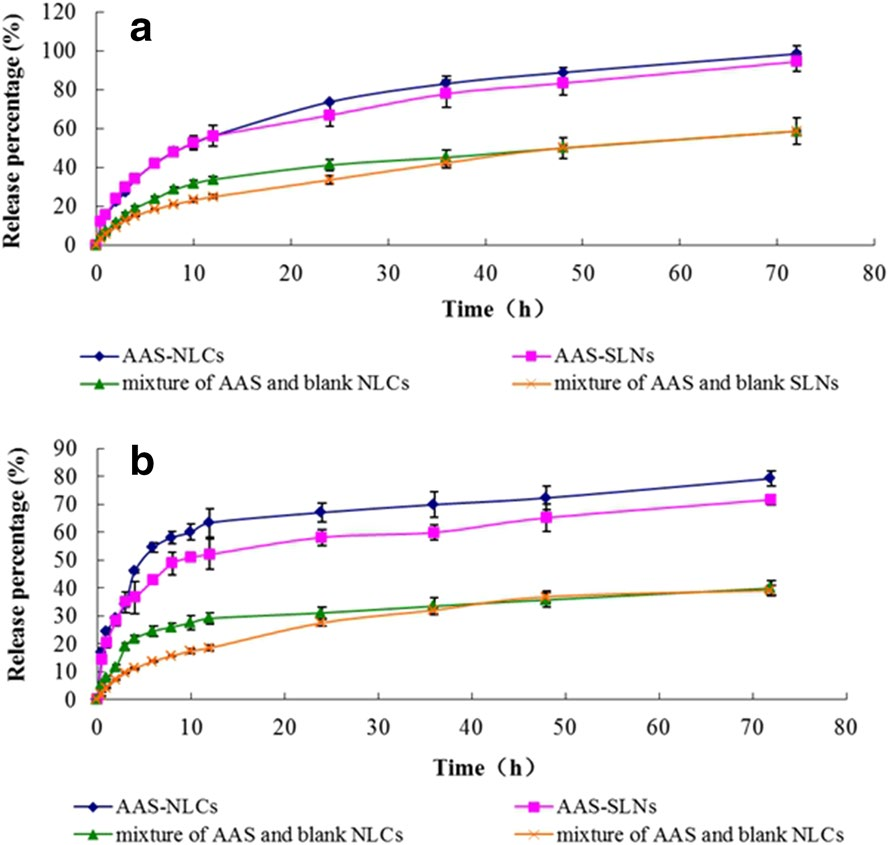
In vitro release profiles. Release of **a** LA and **b** RAN from AAS-NLCs, AAS-SLNs, the mixture of AAS and unloaded NLCs, and the mixture of AAS and unloaded SLNs (n = 3).

The cumulative release percentage profiles of LA and RAN from the AAS-NLCs, AAS-SLNs, and the mixture of AAS and blank NLCs are shown in Figure 5. The aqueous solubility of LA and RAN in the medium was 849.79 ± 36.95 and 582.06 ± 16.27 µg/mL, respectively, and the sink condition was maintained during the release study. Both SLN and NLC dispersions significantly enhanced the in vitro AAS release for 72 h. The results showed the LA and RAN dispersion from SLNs was 94.6 ± 5.2 and 71.8 ± 1.9%, respectively and from the NLCs was 98.5 ± 4.1 and 79.3 ± 2.7%, respectively. In comparison, their physical mixtures showed LA and RAN from SLNs at 58.8 ± 0.7 and 39.2 ± 1.7%, respectively and from NLCs was 58.8 ± 6.9 and 40.0 ± 2.8%, respectively, under the same experimental conditions. This disparity is most likely due to the disordered crystalline state of the drug that exhibited higher solubilization and was selected for nanoparticle formation [9]. In addition, the total drug released from the NLC formulations was slightly greater than that released from the SLN formulations, which was likely caused by the imperfections in NLCs resulting from the incorporation of liquid lipids into solid lipids [19]. Furthermore, AAS-NLCs exhibited an initial fast release of 12.2 ± 0.7 and 17.2 ± 1.4% for LA and RAN, respectively for 0.5 h, which was possibly due to the portion of AAS that was localized at the surface of the nanoparticles.

### Cytotoxicity studies

**Figure 6:**
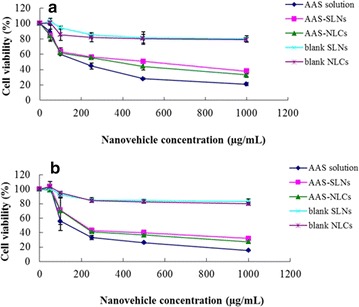
Cytotoxicity of alkaloids isolated from AAS solution, AAS-SLNs, AAS-NLCs, unloaded SLNs and unloaded NLCs. **a** HaCaT cells and **b** CCC-ESF cells (n = 3).

Most lipid nanoparticles formed from glycerides or their derivatives are safe and well tolerated by organisms [20]. The in vitro cytotoxicity of AAS, AAS-SLNs, AAS-NLCs, and the empty vehicles (without AAS, defined as blank NLCs and blank SLNs) was evaluated in HaCaT and CCC-ESF cells. As shown in Figure 6, blank NLCs and SLNs showed no serious cytotoxicity at the tested concentrations (cell survival rates were higher than 80%). The viability of HaCaT and CCC-ESF cells treated with various concentrations of the formulations containing AAS was reduced dose-dependently, and both AAS-SLN and AAS-NLC formulations were significantly (*p* < 0.05) less cytotoxic than the AAS aqueous suspension (1,000 µg/mL) was. These results may be due to the fact that the free drug in the AAS suspension was released and immediately permeated the cells by passive diffusion while the nanoparticles had to be internalized via endocytosis or phagocytosis and released through the drug release process. AAS loaded in the tested nanoparticles achieved a biocompatibility with skin cells that was superior to that of the AAS aqueous suspension.

### Cellular uptake

**Figure 7:**
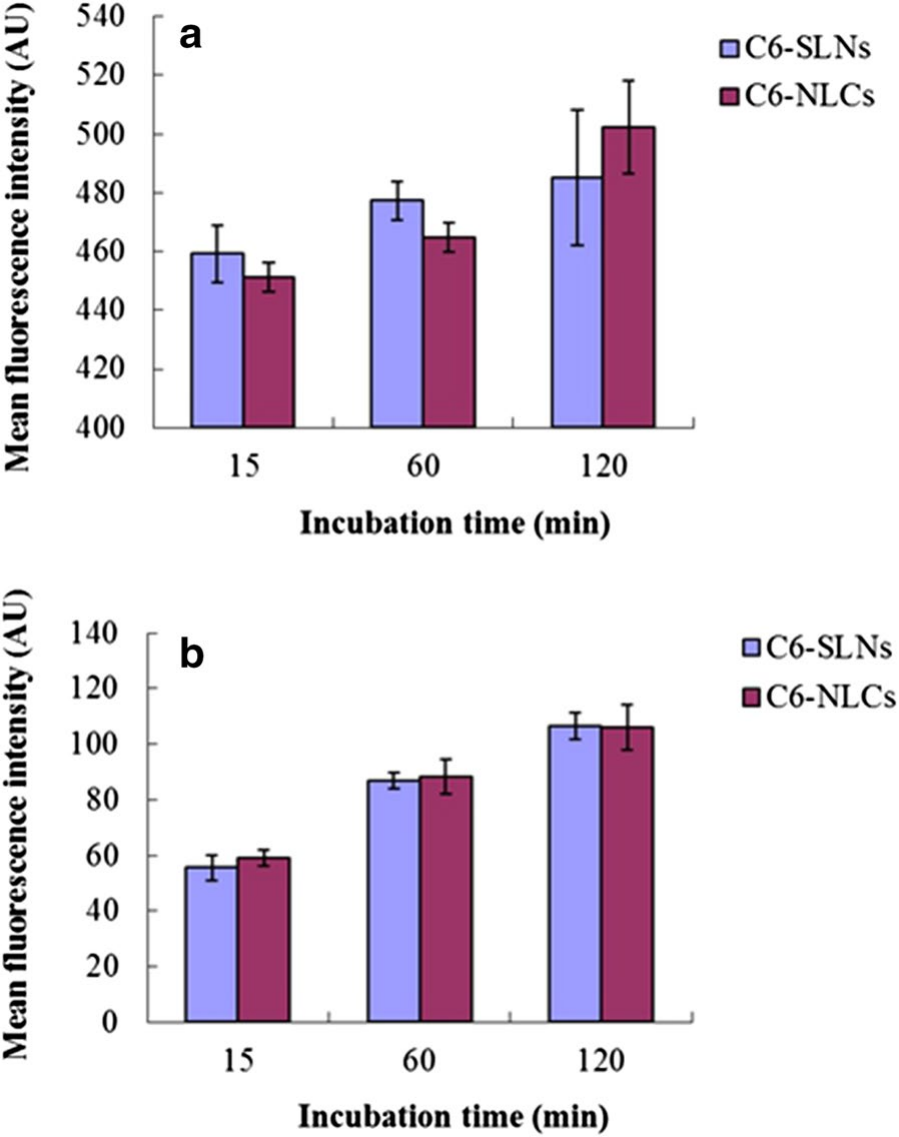
Mean fluorescence intensity of cells treated with coumarin-6 (C6)-labeled NLCs or C6-labeled SLNs. Cells were treated for 15, 60, and 120 min and then measured using flow cytometry. **a** HaCaT and **b** CCC-ESF cells (n = 3).

As shown in Figure 7, HaCaT and CCC-ESF cells showed time-dependent uptake of C6-labeled NLCs and SLNs. Moreover, differences in the fluorescence intensity between the NLC- and SLN-treated cells at the same incubation time were not significant (*p* > 0.05). The similar uptake of NLCs and SLNs in HaCaT and CCC-ESF cells suggests that the solid lipids in the NLCs and SLNs had a similar effect on their biocompatibility.

### Endocytosis pathway

**Figure 8:**
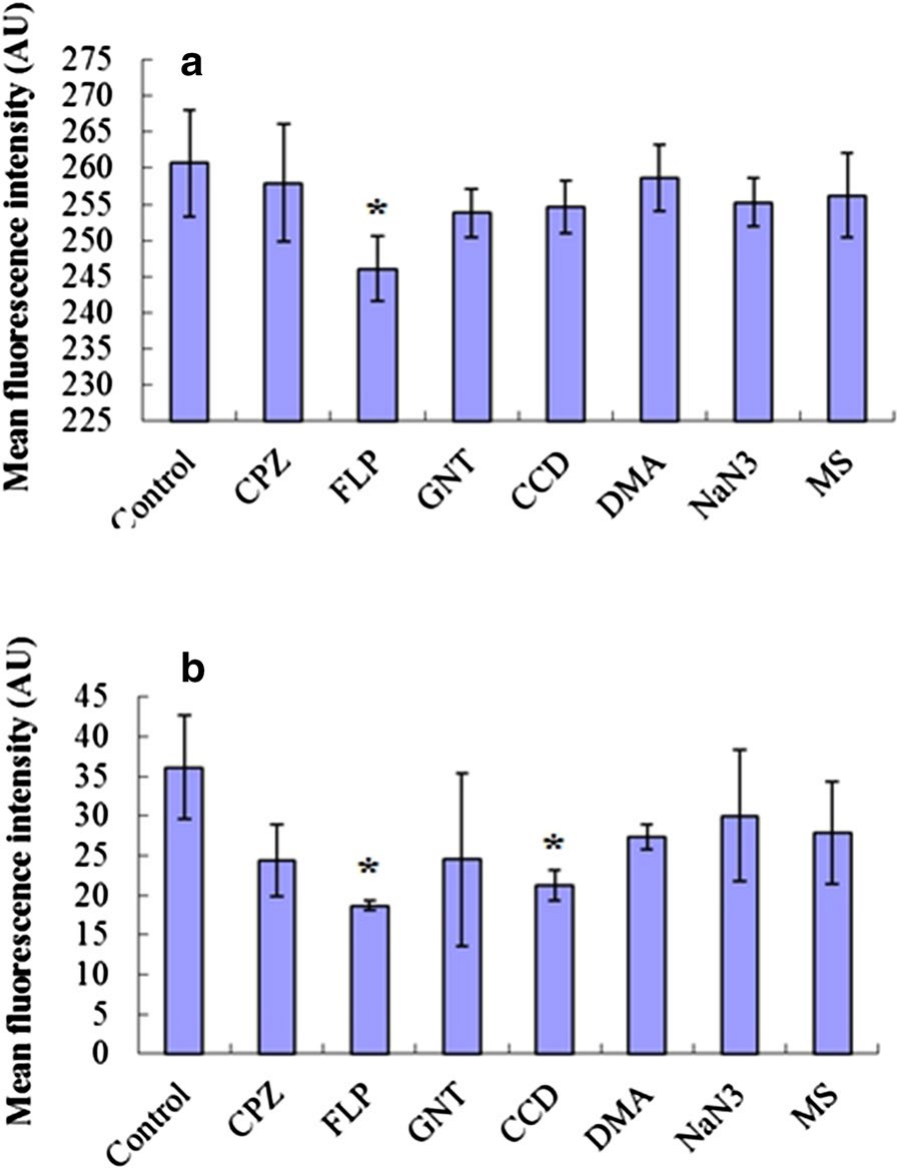
Effects of endocytosis inhibitors on cellular uptake. Effects of inhibitors in **a** HaCaT and **b** CCC-ESF cells (n = 3, *p < 0.05 vs. control group).

To further characterize the endocytosis pathways through which the NLCs deliver drugs, seven endocytosis inhibitors were tested in HaCaT and CCC-ESF cells. These inhibitors included chlorpromazine (CPZ, cationic amphipathic molecule that inhibits clathrin disassembly) [21], filipin III (FLP, inhibitor of caveolae-mediated endocytosis via combination with cholesterol) [22], genistein (GNT, inhibitor of tyrosine kinases associated with caveolae-mediated endocytosis) [23], cytochalasin D (CCD, which disrupts actin filaments and inhibits actin polymerization) [24], amiloride (DMA, blocker of epithelial Na^+^ channels that prevents non-specific macropinocytosis) [25], sodium azide (NaN_3_, a metabolic inhibitor that restrains cellular energy metabolism) [26], and monensin sodium (MS, a Na^+^ ionophore that has been reported to cause a marked increase in intracellular Na^+^ concentration) [27]. As shown in Figure 8, CPZ, GNT, DMA, NaN_3,_ and MS treatments did not significantly affect the internalization of the C6-labeled NLCs in CCC-ESF cells. In contrast, FLP and CCD significantly inhibited the uptake of NLCs in comparison with the control treatment (*p* < 0.05) and the inhibitory effect of ELP was stronger than that of CCD was. This suggests that the uptake of C6-labeled NLCs into CCC-ESF cells was mainly mediated by caveolae-mediated endocytosis but macropinocytosis was also involved. In contrast, of the tested endocytosis inhibitors, only FLP significantly reduced the average fluorescence intensity of the HaCaT cells (*p* < 0.05), showing that caveolae-mediated endocytosis was involved in the internalization of HaCaT cells. These results suggest that the internalization of the drug cargo of NLCs involved cholesterol and lipid rafts in the HaCaT and CCC-ESF cells.

### Confocal imaging

**Figure 9:**
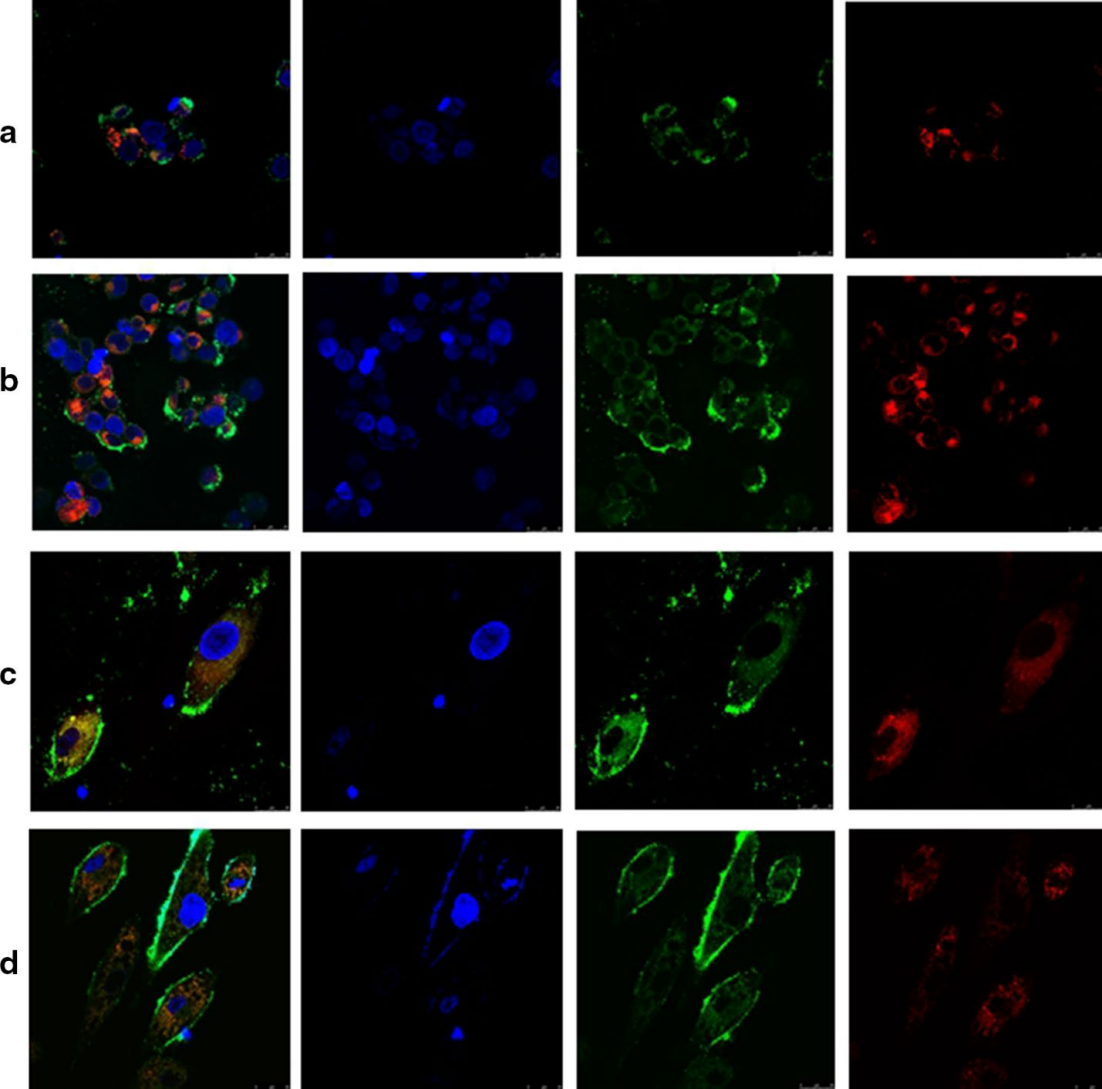
LSCM images. Representative LSCM images of **a** HaCaT cells after incubation with C6-labeled SLNs, **b** HaCaT cells after incubation with C6-labeled NLCs, **c** CCC-ESF cells after incubation with C6-labeled SLNs, and **d** CCC-ESF cells after incubation with C6-labeled NLCs.

The uptake of C6-labeled NLCs and SLNs by the HaCaT and CCC-ESF cells was visualized using LSCM (Figure 9). Hoechst-stained nuclei appeared blue and LysoTracker Red-labeled lysosomes appeared red. The colocalization of C6-labeled NLCs or SLNs (green) and LysoTracker (red) in the periphery of HaCaT and CCC-ESF cells produced yellow fluorescence in the cytochylema. After incubation with C6-labeled nanoparticles, cells showed strong green and weak red fluorescence in the cytosol, which was probably caused by lysosomal escape of C6 from the NLCs, and the LysoTracker lost its fluorescence under neutral conditions in the cytosol [28]. These findings demonstrate that C6-labeled nanoparticles facilitate drug outflow from lysosomes, and that the fluorochrome is primarily distributed in the cytochylema.

### In vitro permeation studies

**Figure 10:**
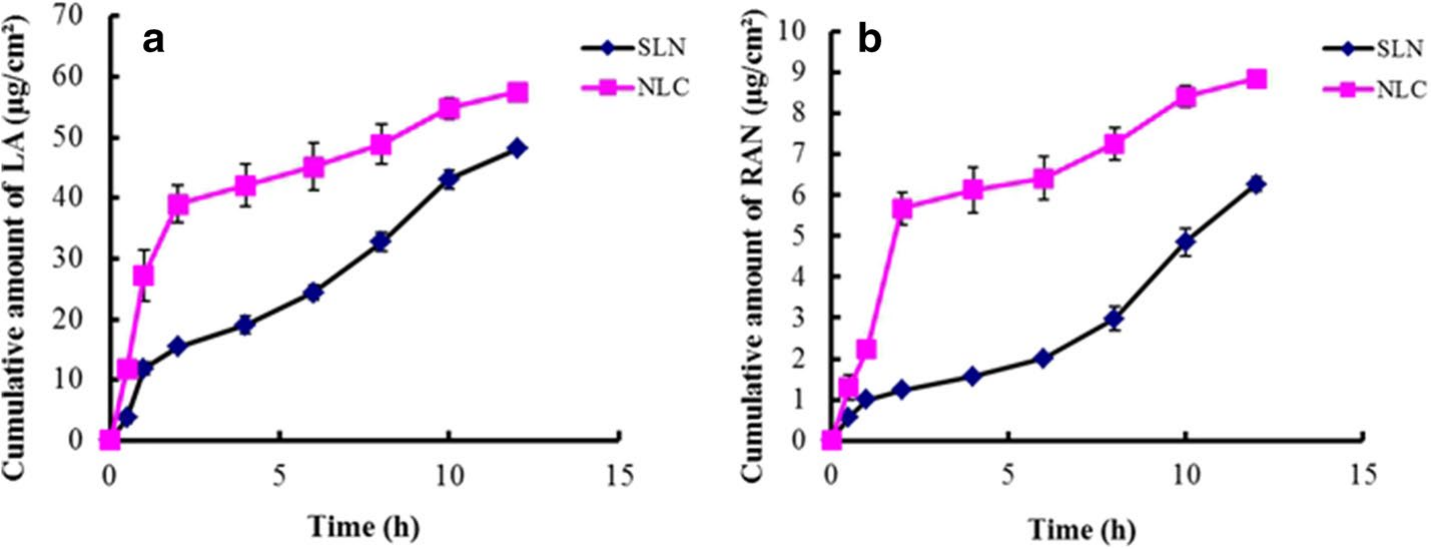
In vitro skin-permeation profiles. Representative profiles of **a** LA and **b** RAN from AAS-NLCs and AAS-SLNs (n = 3).

The enhanced transdermal permeation of drugs packaged in the lipid nanoparticles in comparison with other vehicles is mainly due to the increased surface area in which they interface with skin corneocytes, superior skin occlusion characteristics, and more effective hydration of the stratum corneum (SC) [29]. In our studies, the AAS-NLCs enhanced the transdermal penetration of LA and RAN (Figure 10) more effectively than the AAS-SLNs did. Initially, AAS was released rapidly from both SLNs and NLCs but showed sustained release afterward, which was consistent with the in vitro release study. Over the duration of the experiment, NLCs released cumulative amounts of LA and RAN that were greater than the amounts released by the SLNs. The enhanced drug release from the NLC formulations may be attributable to the greater drug encapsulation achieved with the NLC vehicle. In addition, labrasol, which was used in the NLCs, acts as a surfactant, which may loosen or fluidize the lipid bilayers of the SC [19].

### Nanoparticle tracking analysis (NTA)

**Figure 11:**
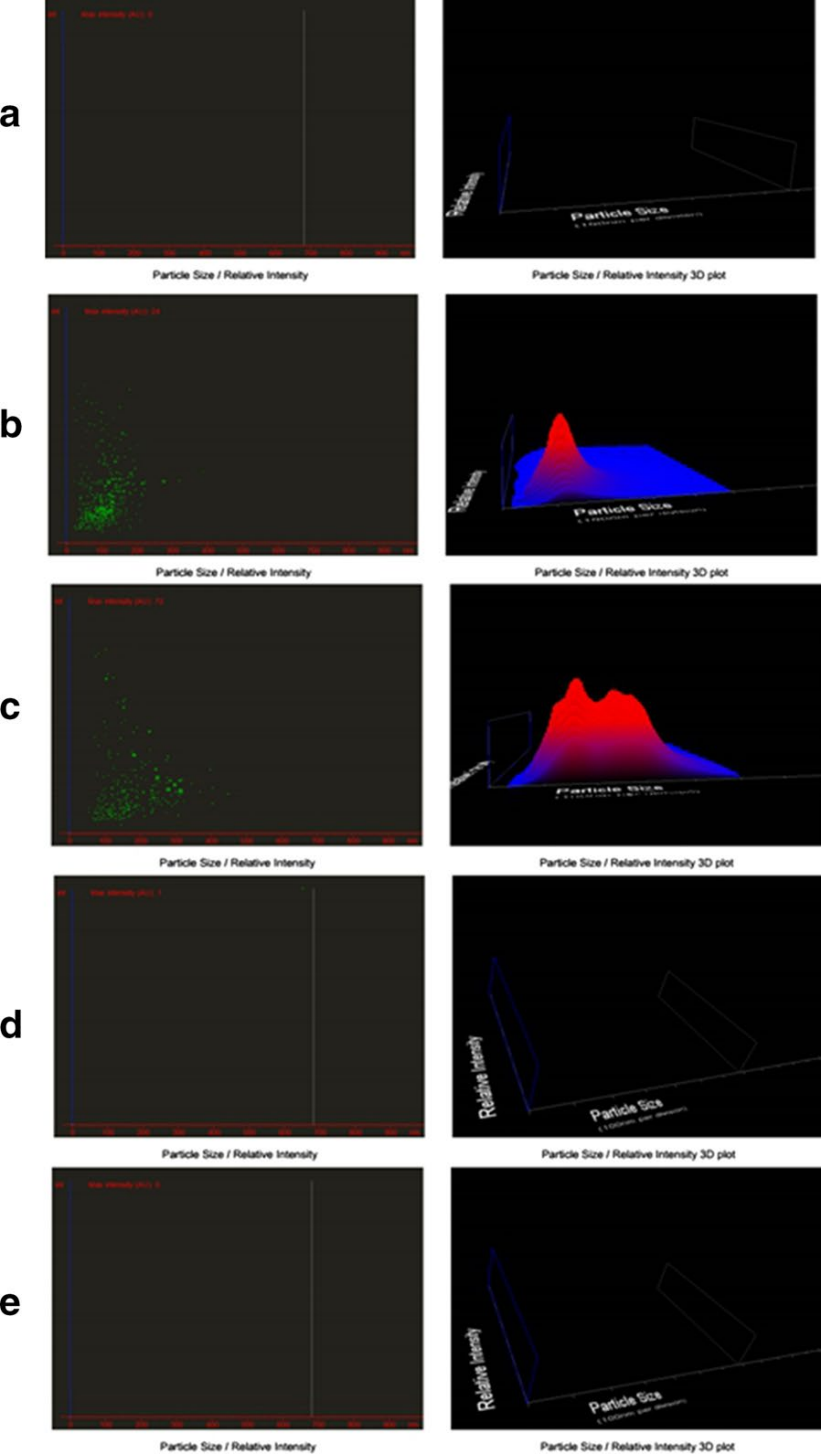
NanoSight image of test formulations. Representative images of **a** blank control, **b** C6-labeled NLCs and **c** C6-labeled SLNs diluted 1,000,000-fold, **d** a sample treated with C6-labeled NLCs, and **e** a sample treated with C6-labeled SLNs.

No fluorescent nanoparticles were found in the blank control sample (Figure 11a), demonstrating that flaking skin particles did not influence the experimental outcome. The C6-labeled NLCs and SLNs were each diluted 1,000,000-fold and fluorescent nanoparticles were detected (Figure 11b, c). This ensured the sensitivity of the method for small quantities of nanoparticles such as those that might be transported across the skin. As shown in Figure 11d and e, fluorescent nanoparticles were not detected in the receptor fluid after permeation by C6-labeled NLCs or SLNs. It is also believed that rigid particles above 10 nm do not penetrate intact skin [30]. This finding is consistent with reports on lipid nanoparticles, which are not considered to penetrate the SC [14].

### Effect on SC structure

**Figure 12:**
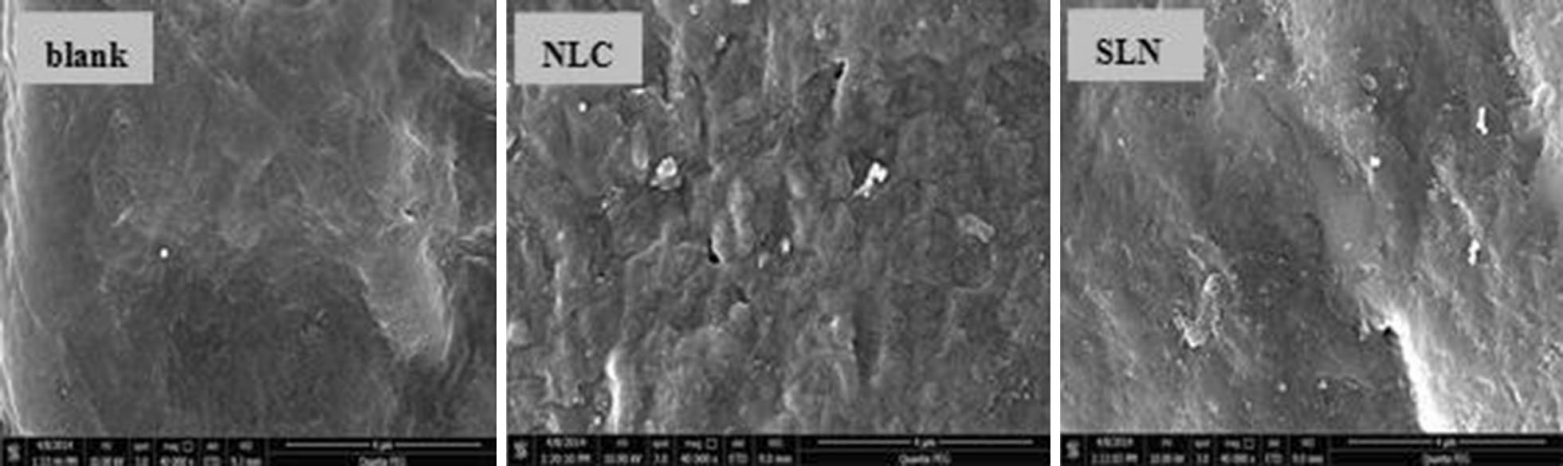
Microstructures of nude mouse skin. Skin samples with normal control (blank), AAS-NLCs-treated (NLC), and AAS-SLNs-treated (SLN) obtained via SEM (×400,000 magnification).

The surface of blank nude mouse skin (without any treatment) was intact, smooth, and showed a tightly compacted SC layer (Figure 12). However, the surface of skin exposed to the NLC or SLN formulations was jagged and the cracks in the SC were larger in size (Figure 12). The lipids and surfactants in the vehicles may erode the SC, and thereby disrupt its lipid arrangement. This disruptive effect on the SC may have been exacerbated by the occlusive effect of the nanoscale lipid particles.

### In vivo skin microdialysis

In the in vitro and in vivo RR validation studies, STD1–STD6 were used as perfusates and a linear correlation was obtained between the drug concentrations in the perfusate and the dialysate (LA, [C_p_–C_d_] = 0.431C_p_ − 0.022, r^2^ = 0.997 [in vitro]; [C_p_–C_d_] = 0.345C_p_ + 0.215, r^2^ = 0.991 [in vivo]; RAN, [C_p_–C_d_] = 0.418C_p_ + 0.394, r^2^ = 0.996 [in vitro]; [C_p_–C_d_] = 0.318C_p_ + 0.330, r^2^ = 0.987 [in vivo]). This correlation implied that in vitro and in vivo RR measurements obtained via retrodialysis are independent of perfusate concentrations, validating the feasibility of the microdialysis method to detect true unbound extracellular levels of LA and RAN.

**Figure 13:**
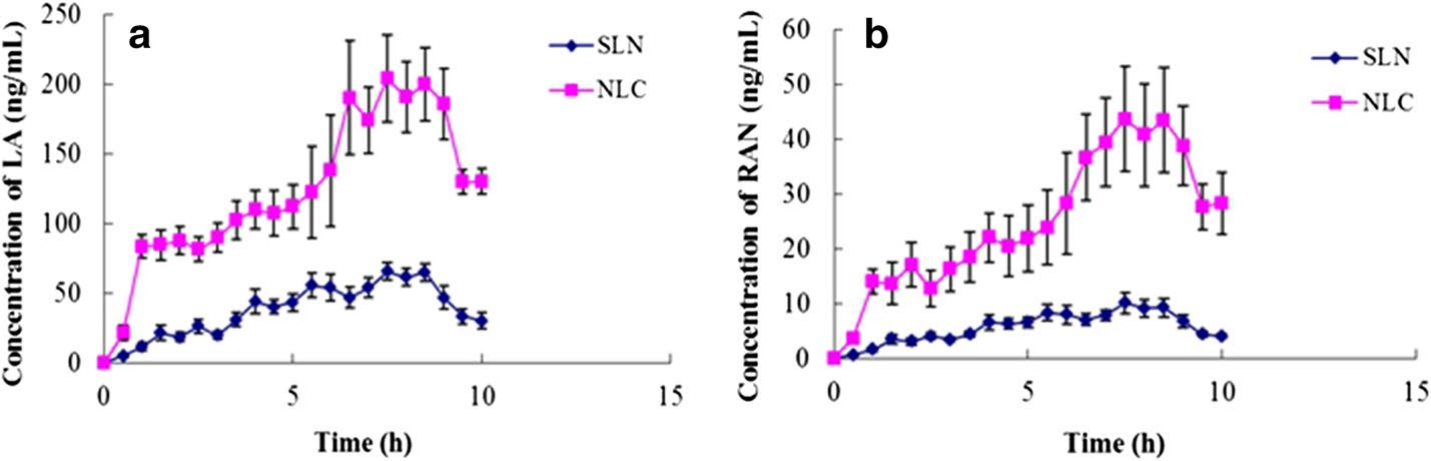
Time course of rat skin penetration by test alkaloids. **a** LA and **b** RAN concentrations sampled after the administration of AAS-NLCs and AAS-SLNs on the abdominal skin of rats over a period of 10 h (n = 5).

In vivo time-concentration profiles of LA and RAN after topical application of the AAS-NLCs and AAS-SLNs on the abdominal skin in rats are depicted in Figure 13, and results for some pharmacokinetic parameters are summarized in Table 3. The peak concentration (C_max_) of the AAS-NLC and AAS-SLN formulations were similar at approximately 8 h after administration. The drug concentrations at the sites where NLCs or SLNs were applied first increased and then decreased, which implied similar patterns of transdermal drug absorption for both lipid nanoparticle formulations. Their occlusive effects and ability to hydrate the SC [31] enhance the transdermal drug permeation achieved by NLCs and SLNs. Drug absorption may be enhanced by the formation of a lipid membrane that fuses with the SC and allows the drug to diffuse into the skin. Moreover, drug molecules must be released from nanoparticles before they can be absorbed, which may cause drug levels in the skin to fluctuate. The higher C_max_ values (LA and RAN, 207.45 ± 29.89 and 46.60 ± 9.56 ng/mL, respectively) were obtained with AAS-NLCs, and the area under the concentration–time curve (AUC_0–t_) of LA and RAN was 3.27- and 4.42-fold higher, respectively than that of AAS-SLNs (Table 3). Altering skin physiology and modifying NLC formulations to influence drug partitioning and solubility are potential strategies to enhance transdermal drug penetration. The addition of labrasol as a liquid lipid increased the drug-loading capacity of NLCs and fluidized the lipid bilayers of the SC, which may enhance the permeation effect compared with that of the SLNs [19].

**Table 3:** Pharmacokinetic parameters of LA and RAN released from AAS-NLCs and AAS-SLNs in rat dermis measured using in vivo microdialysis

Parameter	AAS-NLCs		AAS-SLNs	
	LA	RAN	LA	RAN
T_max_ (h)	7.70 ± 0.84	8.00 ± 0.50	7.70 ± 0.45	7.50 ± 0.00
C_max_ (ng/mL)	207.45 ± 29.89*	46.60 ± 9.56*	65.93 ± 6.37	10.07 ± 1.93
MRT_0–t_ (h)	6.04 ± 0.03	6.24 ± 0.07	6.11 ± 0.05	6.04 ± 0.07
AUC_0–t_ (ng/mL × h)	1,239.07 ± 176.39*	248.44 ± 50.85*	378.72 ± 56.04	56.16 ± 8.89

Mean ± SD, n = 5, * *p* < 0.05 vs. AAS-SLNs.

## Conclusion

In this study, AAS-NLCs were successfully prepared with the following components in optimal proportions: Precirol ATO5, labrasol, Cremophor RH40, and AAS (6.7, 2.3, 5.5, and 0.9%, w/v, respectively). The NLCs strongly affected cholesterol and lipid rafts in the cellular pathway. These results show that the NLCs achieved transdermal delivery of AAS that was superior to that produced by SLNs, suggesting that NLCs are a potentially suitable transdermal vehicle for AAS delivery.

## Methods

### Materials

LA and RAN (purity >98%, each) were purchased from Shaanxi Chenguang Pharmaceutical Co., Ltd. (Xi’an, China). AS was purchased from Shaanxi Minsheng Medicine Co., Ltd. (Xi’an, China). Caprylocaproyl macrogol-8 glycerides (Labrasol^®^) and glyceryl palmitostearate (Precirol^®^ ATO 5) were provided by Gattefossé (Montesquieu, France). Polyoxyl castor oil (Cremophor^®^ RH40) was obtained from BASF (Ludwigshafen, Germany). High-glucose Dulbecco’s modified Eagle medium (DMEM-high) was obtained from Thermo Fisher Scientific (Waltham, MA, USA). Trypsin (0.25%), 0.02% ethylenediaminetetraacetic acid (EDTA), fetal calf serum (FCS), 3-(4,5-dimethylthiazol-2-yl)-2,5-diphenyl tetrazolium bromide (MTT), and phosphate-buffered saline (PBS) were obtained from Shanghai Usen Biotechnology (Shanghai, China). Filipin III, genistein, cytochalasin D, 5-(*N*,*N*-dimethyl)-amiloride hydrochloride, monensin sodium, Lyso-Tracker^®^ Red DND-99, and Hoechst33258 stain were purchased from Santa Cruz Biotechnology, Inc. (Santa Cruz, CA, USA). Sodium azide was obtained from Beijing Bei-na Kai-chuang Biotech Co., Ltd (Beijing, China), and chlorpromazine hydrochloride was obtained from Aladdin Industrial Inc. (Shanghai, China). All other chemicals were obtained from Sinopharm Chemical Reagent (Shanghai, China) and were of high-performance liquid chromatography (HPLC) or analytical grade.

### Animals and cell lines

Male Sprague–Dawley rats and nude mice weighing 200 ± 20 and 25 ± 5 g, respectively were used in this study, which was conducted with the approval of the Animal Ethical Committee of the Shanghai University of Traditional Chinese Medicine (permit SYXK [Hu] 2009-0069). All the animals were acclimatized at a temperature of 25 ± 2°C and relative humidity of 70 ± 5% for 1 week with food and water provided ad libitum. The HaCaT cell line was purchased from the American Type Culture Collection (ATCC, Manassas, VA, USA), and the CCC-ESF cell line was obtained from the Chinese Academy of Medical Sciences (Beijing, China).

### HPLC analysis

LA and RAN were analyzed using an Agilent HPLC system (HP 1260, Agilent Technologies, Inc., Santa Clara, USA) with an octadecylsilyl column (Inspire™ C18 (Dikma Technologies, Beijing, China), 250 mm × 4.6 mm, 5 µm). The mobile phase consisted of a solution of methanol and 0.01 mol L^−1^ monosodium phosphate aqueous solution (80:20, v/v) at a flow rate of 1.0 mL min^−1^. The column temperature was maintained at 30°C and the detector was set at 252 nm. Quantified samples were filtered through a 0.45-µm filter membrane before automatic injection into the HPLC system.

### Preparation of AAS

AAS was extracted using alcohol reflux extraction methods. Briefly, powdered AS was soaked in acidified water (pH 1)/90% ethanol (v/v) at a ratio of 1:10 (w/v), and then extracted by reflux with heating for 2 h in a water bath at 100°C. The extract was collected, and then replaced with fresh solvent. This extraction procedure was repeated twice. After filtration and alkalization (pH > 10) with ammonium hydroxide, the extracts were further treated with chloroform and concentrated by vacuum distillation. To prepare the AAS, the residue was dissolved in acetone and recrystallized with diethyl ether, and the obtained extracts were vacuum-dried (relative vacuum, −0.1 MPa; temperature: 60°C) for 12 h. The main components of AAS, which have superior pharmacological effects were LA and RAN (yield, LA 69.47 and RAN 9.16%, w/w, respectively).

### Ultra-performance liquid chromatography (UPLC)-tandem mass spectrometry (MS/MS) analysis

The in vivo dialysate samples were analyzed using a Thermo Ultimate 3000 ultra-performance liquid chromatography system coupled to a Thermo TSQ Quantum triple-quadrupole tandem mass spectrometer (Thermo Finnigan, San Jose, CA, USA) equipped with an electrospray ionization source. The Xcalibur 1.2 data analysis system was used. Chromatographic separation was achieved on an octadecylsilyl column [Hypersil GOLD™ C18 (Thermo Finnigan, San Jose, CA, USA), 100 mm × 2.1 mm, 5 µm] maintained at 30°C. The mobile phase consisted of methanol:water:acetic acid (70:30:0.2, v/v/v) with a flow rate of 0.2 mL/min, and the injection volume was 5 µL. The mass spectrometer was operated in the positive mode. The spray voltage was set to 3,500 kV and the capillary temperature was set at 350°C. Nitrogen was used as the sheath (35 psi) and auxiliary gas (10 psi). The precursor-to-production transitions were monitored at *m/z* 585 → 356 for LA and *m/z* 601 → 422 for RAN using the selected reaction monitoring mode. Quantified samples were extracted with diethyl ether and dissolved in methanol before automatic injection into the UPLC-MS/MS.

### Preparation of AAS-NLCs

The different AAS-NLC formulations were prepared using the hot-melt high-pressure homogenization method [32] with Precirol ATO 5, labrasol, and Cremophor RH40 as the solid lipid, liquid lipid, and surfactant, respectively. Briefly, AAS was dissolved in the solid (Precirol ATO 5) and liquid (labrasol) lipids, which were melted in a water bath at 75°C. The required amount of the aqueous phase (Cremophor RH40 in double-distilled water) was then added to the oleic phase dropwise under high-speed mixing using a disperser (Ultra Turrax T25, IKA, Staufen, Germany). The resulting pre-emulsion was then immediately passed through a high-pressure homogenizer (NS1001 L, GEA, Parma, Italy). To optimize the NLC formulations, a uniform design with a U*7(7^4^) table was used in this study that included the following factors: the amount of lipids (solid and liquid lipids, factor A), the amount of surfactant (factor B), the ratio of solid lipids to liquid lipids (factor C), and the ratio of drugs to lipids (factor D). This scheme produced seven NLC formulations (NLC1–NLC7) that were characterized with regards to particle size, EE, and drug DL. The AAS-SLN preparations used for comparison were prepared using the same method with Precirol ATO5 (9.0%, w/v), Cremophor RH40 (5.5%, w/v), and the same drug content.

### Characterization of AAS-NLCs

#### Particle size and zeta potential (ZP)

A computerized Malvern Autosizer Nano ZS90 inspection system (Malvern Instruments, Malvern, UK) was used to measure the average particle size and zeta potential (ZP) of each preparation via dynamic light scattering. The samples were diluted with distilled water before measurements were taken and each was performed in triplicate.

#### EE and DL

The free drug was separated using an ultrafiltration method [33]. Centrifugal filter tubes (10 kDa, Pall Corporation, Port Washington, NY, USA) were used to estimate the EE and DL. Nanoparticle suspensions (0.5 mL) were placed in the centrifugal filter tubes, and centrifuged for 15 min at 5,000 rpm. The free drug was separated from the drug entrapped in the nanoparticles and collected at the bottom of the tube through the membrane. The separated and total drug contents were evaluated using a validated HPLC. The total drug content in each preparation was calculated after extraction with acetonitrile in an ultrasonic bath. The EE and DL were calculated using the following equations:

1 $${\text{EE}}\% \, = \, \left( {W_{total} - W_{free} } \right)/W_{total} \times \, 100$$

2 $${\text{DL}}\% \, = \, \left( {W_{total} - W_{free} } \right)/W_{lipids} \times \, 100$$

where, *W*_*total*_, *W*_*free*_, and *W*_*lipids*_ are the total amount of LA and RAN in the preparation, the amount of untrapped LA and RAN, and the amount of lipids used in the formulation, respectively.

#### Tem

The physical appearance of the AAS-NLCs were examined and with that of the AAS-SLNs using TEM (JEM-1230; Jeol, Ltd., Tokyo, Japan). The samples were prepared for negative staining as follows. The samples were diluted with distilled water and placed on a film-coated copper grid (Zhong Jing Ke Yi Technology Inc., Beijing, China) to dry for about 20 min. Next, a drop of 2% phosphotungstic acid was added to the film and allowed to dry for 10 min, after which the sample was examined under the TEM.

#### Dsc

DSC was performed using a differential scanning calorimeter (Shimadzu DSC-60; Shimadzu Corporation, Kyoto, Japan). The sample (lyophilized in advance using mannitol 10%, w/v as the cryoprotectant) was heated at the scanning rate of 10°C per min over a temperature range of 30–300°C. An empty aluminum pan was used as a reference for comparison.

#### Xrd

XRD analysis of the AAS-loaded lipid nanoparticles (lyophilized in advance using mannitol 10%, w/v as the cryoprotectant) was performed using an X-ray diffractometer (Rigaku Corporation, Tokyo, Japan) to assess their crystalline structures. The X-ray diffraction patterns were recorded at 2θ values of 3–50° at a scanning speed of 5° per min using a Cu-Kα radiation source.

#### In vitro release

The dialysis method was used to assess the in vitro drug release pattern [34]. Dialysis bags (molecular weight cutoff, 14 kDa, Pall Corporation, Port Washington, NY, USA) containing 5 mL of the test formulations were firmly tied and immersed in 100 mL of normal saline containing 20% polyethylene glycol 400 (PEG 400, v/v) at 37 ± 0.5°C and stirred at 120 rpm with a paddle. At each sampling time point, 1 mL of the sample was withdrawn, and then replaced with the same volume of fresh medium. The withdrawn samples were measured using HPLC. All experiments were performed in triplicate.

### In vitro cell uptake

#### Cell culture

HaCaT and CCC-ESF were grown in fresh culture medium (DMEM-high) containing 10% FCS (v/v) and maintained at 37°C under humidified conditions with 5% CO_2_ (Forma 3111, Thermo Fisher Scientific). For the subculture, cells growing as a monolayer were detached from the culture dish with 0.05% (w/v) trypsin solution. All cell-handling procedures were performed on a super-clean bench (VCM-620; Dabao Instrument, Suzhou, China) and aseptic techniques were employed.

#### Cytotoxicity

The cytotoxicity of free drugs, AAS-NLCs, AAS-SLNs, and empty vehicles was determined using the MTT assay [35]. The AAS solution was prepared using dimethylsulfoxide solution and culture medium (DMEM-high) while the AAS-NLC preparations, AAS-SLN preparations, and empty vehicle suspension were prepared in culture medium (DMEM-high) over a range of 50–1,000 µg/mL. The working solutions at various concentrations were added to cells cultured in 96-well microplates at 5 × 10^3^ cells/well and incubated for 4 h. Then, the culture fluid was replaced with fresh DMEM (no FBS) containing 500 μg/mL MTT. After a 4-h incubation at 37°C, the culture medium was removed and replaced with 200 μL dimethylsulfoxide. The plate was shaken for 10 min in the dark and the absorbance was determined at 570 nm in a microplate reader (Biotek Synergy HT, Gene Company Limited, Hong Kong, China). All measurements were performed in triplicate and results were calculated as a percentage of the values obtained from the control wells (untreated cells).

#### Cellular uptake

Cellular uptake was evaluated using flow cytometry with coumarin-6 (C6) dehydrated alcohol solution (1.0 mg/mL) instead of AAS. NLC and the compared SLN formulations containing 10 µg/mL C6 were prepared. Cells were incubated with the C6-labeled formulations in the culture dish for 15, 60, and 120 min, after which the culture medium was removed and the cells were rinsed thrice with 1 mL PBS (pH 7.4). After trypsinization, the cells were carefully collected into 5-mL BD tubes (Falcon 352002; Becton, Dickinson and Company, Franklin Lakes, NJ, USA) and centrifuged at 1,500 rpm at 25°C in a refrigerated centrifuge (Thermo Fisher Scientific, Waltham, MA, USA). The supernatant was removed and the cells were resuspended in 0.5 mL of PBS (pH 7.4, containing 1% FCS, v/v) in the BD tubes. A total of 20,000 cells were analyzed using a flow cytometer (FCM, Becton, Dickinson and Company, Franklin Lakes, NJ, USA). Cells that had not been incubated with any fluorophore served as the blank control cells.

#### Endocytic pathway

Endocytosis mechanisms were assessed using cellular uptake assays in the presence of chemicals known to inhibit specific endocytosis pathways [36]. Cells were incubated with CPZ, FLP, GNT, CCD, DMA, NaN_3_, or MS (at 10, 5, 54, 1.52, 2.66, and 2.08 µg/mL, respectively) for 30 min at 37°C. After exposure to these inhibitors, 200 µL of C6-labeled NLCs was added to each well and incubated for 30 min. After the incubation, cells were prepared for flow cytometry as described earlier and the data for at least 20,000 cells were acquired. The cells treated with C6-labeled NLCs alone were considered as the control cells.

#### Confocal imaging

First, 100 µL of the culture medium (no FCS) in glass-bottom culture dishes (MatTek P35G-0-10-C35 mm, MatTek Corporation, Ashland, USA) was removed and replaced with an equal volume of either the C6-labeled NLC suspension or the C6-labeled SLN suspension. After a 30-min incubation in 5% CO_2_ atmosphere maintained at 37°C, the culture fluid was removed, and the culture dish was washed thrice with 1 mL pre-cooled PBS (pH 7.4). Fresh DMEM (no FBS) containing 50 nM LysoTracker Red was added to each sample and the cells were incubated for 30 min at 37°C. Hoechst 33342 was added to a final concentration of 10 μg/mL and the cells were cultured for a further 5 min at 37°C. The culture fluid was removed rapidly, 1 mL of aqueous paraformaldehyde solution (4%, w/v) was added to the culture dish to fix the cells for 15 min, the fixative was removed, and then the cells were washed thrice with 1 mL PBS (pH 7.4). The cells were optically scanned within 2 h at different increments through the z-axis with a TCS SP5 confocal imager (Leica Microsystems, Wetzlar, Germany).

### In vitro transdermal studies

Rats were anesthetized, euthanized, their abdominal fur was removed with a razor, and then the skin was excised carefully and washed with normal saline. In vitro permeation experiments were conducted using a Franz diffusion cell (Fulansi Electronic Science and Trade Co, Ltd, Tianjin, China) fitted with excised rat skin [37]. Each donor compartment had a diffusion area of 2.0 cm^2^ and each receptor compartment was filled with 12.5 mL of freshly prepared normal saline containing 20% PEG 400 (v/v) to provide sink conditions [38]. The saline in the receptor compartment was maintained at a temperature of 37 ± 0.5°C and stirred at 500 rpm using a magnetic bar. One mL of each formulation was added to the donor compartment, and it was sealed with parafilm. At predetermined time points, 1 mL of the sample was removed from the receptor compartment, and replaced with an equal volume of receptor fluid equilibrated to 37 ± 0.5°C. The permeation studies were performed in triplicate and the collected samples were analyzed using HPLC.

### In vitro fluorescence detection-based NTA in receptor fluid

A Franz diffusion cell fitted with excised rat skin was prepared as described earlier. The receptor compartment was filled with purified water containing 0.02% NaN_3_ and the donor compartment was filled with 1.0 mL of the C6-labeled NLC suspension, C6-labeled SLN suspension, or 1.0 mL of C6 suspended in distilled water as a blank control sample. The diffusion cell was stirred at 500 rpm while the temperature was maintained at 37 ± 0.5°C. After 12 h, samples were removed from each receptor compartment and measured using a NanoSight NS300 system (Malvern Instruments, Malvern, UK), which was equipped with a scientific sCMOS camera, a 405 nm laser with temperature control, and a 503 nm long-pass filter for fluorescent particle analysis. NanoSight NS300 NTA 3.0 software was used for the data collection and the temperature was maintained at 25°C throughout the experiment.

### Effect on SC structure

A nude mouse was anesthetized and immobilized on a mat with the abdomen facing upward, and then the skin in this region was divided into three areas. A flat cylindrical plastic cover with an area of 1 cm^2^ that served as a drug pool was glued above each skin area using cyanoacrylate adhesive. One mL of NLCs or SLNs was applied to the plastic cover, and the untreated skin area was maintained as a control region. After 8 h, the mouse was euthanized, and the abdominal skin was collected and washed with normal saline. The skin samples were fixed in glutaraldehyde for 24 h, rinsed with PBS (pH 7.4), fixed in 1% osmic acid, dehydrated with ethanol, and then dried using CO_2_ critical point drying. Finally, the skin was sputtered with platinum and evaluated using a scanning electron microscope (SEM, Quanta FEG250, FEI, Hillsboro, OR, USA).

### Microdialysis system

The microdialysis system used was composed of a WZ-50C6 microinfusion pump (Smiths Medical, St. Paul, MN, USA) with a 20-mL plastic syringe and a linear microdialysis probe. The regenerated cellulose (inner diameter, 200 µm; outer diameter, 280 µm; molecular weight cut-off, 13 kDa) was used to prepare Spectra/Por^®^ microdialysis hollow fibers (Spectrum Laboratories, Inc., Houston, TX, USA). The linear microdialysis probes were made by gluing the fibers to the quartz capillary tubing (Welch Materials, Inc., Shanghai, China) using cyanoacrylate adhesive (Mxbon^®^ Super Glue, Cartell Chemical Co., Ltd., Chia-Yi Hsien, Taiwan). The inlet tubes of the probes were connected to the microinjection pump using polyethylene tubing [39]. In all experiments, the length of the membrane accessible to dialysis was 20 mm, and the perfusate flow rate was 0.2 mL/h. Cannulas were used as insertion guides and vials were used to collect the dialysate samples.

Standard (STD) solutions of LA and RAN were prepared by dissolving pure LA and RAN in normal saline containing 20% PEG400 (v/v). The concentrations of the STD solutions (STD1–STD6) were 1, 5, 10, 25, 50, and 100 ng/mL, respectively.

### In vivo recovery validation in vivo correction

Before the microdialysis studies, in vitro recovery was estimated to ensure that the probes would provide reproducible and efficient sampling. The dialysis membrane portion of the linear probe was completely submerged in a standard solution of LA or RAN (STD, prepared by dissolving pure LA or RAN in 20% PEG 400 in normal saline, v/v) at 37°C in a 50-mL beaker. The probe was perfused with 20% PEG 400 in normal saline (v/v) at a flow rate of 0.2 mL/h. After an equilibration period of 30 min, the dialysate was collected in a small vial for 30 min and dialysate samples were analyzed using UPLC-MS/MS. Relative recovery (RR_*d*_) was calculated as the slope of the linear regression of the drug concentration in the dialysate (C_*d*_) as a function of the drug concentration in the medium (C_*m*_):

3 $${\text{RR}}_{d} = C_{d} /C_{m} \times { 1}00$$

For the retrodialysis studies, the probe was perfused with STD solutions (C_*p*_) and the medium was replaced with 20% PEG 400 in normal saline (v/v). The drug concentration in the dialysate (C_*d*_) was determined, and RR_*rd*_ was calculated by the following equation:

4 $${\text{RR}}_{rd} = \, \left( {C_{p} {-}C_{d} } \right)/C_{p} \times \, 100$$

In vivo RR was estimated using the retrodialysis-by-drug method. A linear probe was inserted into the dermis of the abdominal skin of a rat anesthetized with intraperitoneal urethane aqueous solution (1.3 g/kg). The rat was perfused with 20% PEG 400 in normal saline (v/v) for 1 h, followed by an equilibration period of 30 min using the STD solution as the perfusate. After a further 30 min of STD perfusion, dialysate samples were collected. UPLC-MS/MS assays were conducted to determine the diffusive loss of the drug indirectly, and RR was calculated using Eq. (4).

### In vivo microdialysis studies

To determine the concentration of the administered drug, a probe was inserted into the dermis of the abdominal skin of a rat anesthetized with intraperitoneal urethane aqueous solution (1.3 g/kg), and the active dialysis window was placed below the site of topical drug administration. The rat was immobilized on a mat with the abdomen facing upward and the fur in this region was manually shaven, taking care not to damage the skin. The ambient temperature was maintained at 25°C. For the in vivo microdialysis sampling following probe implantation, the connective tubing from the probe was secured to the skin with adhesive tape to fix its position. The probe was continuously perfused with 20% PEG 400 in normal saline (v/v) at a flow rate of 0.2 mL/h. The skin was allowed to equilibrate for 1 h before a blank sample was taken, and 1 mL of AAS-NLCs or AAS-SLNs was applied 1.5 h after the start of perfusion. A flat cylindrical plastic cover of about 1 cm in height and 1.5 cm in diameter, with an edge width of 2 mm was glued above the application site using cyanoacrylate adhesive, and the drug was applied to the cover the skin just above the probe. During the experiment, the application site, and the probe were kept at the same level. Dialysate samples were collected in small vials, which were replaced every 30 min. Dialysis sampling was performed for 10 h.

### Statistical analysis

Results are presented as mean ± standard deviation (SD). Differences were analyzed using the Student’s *t* test with statistical program for the social sciences (SPSS) software version 19.0 (IBM Corp., Armonk, NY, USA). P values <0.05 were considered to be statistically significant. The uniform design with a U*7(7^4^) table was optimized using the SPSS software and VB software version 6.0 (Microsoft Corp., Redmond, WA, USA). The cutaneous pharmacokinetic parameters of LA and RAN were calculated using noncompartmental analysis with the DAS 2.0 software (Mathematical Pharmacology Professional Committee of China, Shanghai, China).

## Abbreviations

NLCs: nanostructured lipid carriers
SLNs: solid lipid nanoparticles
LA: lappaconitine
RAN: ranaconitine
AAS: total alkaloids isolated from *Aconitum sinomontanum*
AAS-NLCs: nanostructured lipid carriers loaded with total alkaloids isolated from *Aconitum sinomontanum*
AAS-SLNs: solid lipid nanoparticles loaded with total alkaloids isolated from *Aconitum sinomontanum*
LSCM: laser scanning confocal microscope
FACS: fluorescence-activated cell sorting
PDI: polydispersity index
ZP: zeta potential
EE: entrapment efficiency
DL: drug loading
TEM: transmission electron microscopy
DSC: differential scanning calorimetry
XRD: X-ray diffraction
NTA: nanoparticle tracking analysis
SEM: scanning electron microscope
SC: stratum corneum
CPZ: chlorpromazine
FLP: filipin III
GNT: genistein
CCD: cytochalasin D
DMA: 5-(*N*,*N*-dimethyl)-amiloride hydrochloride
NaN_3_: sodium azide
MS: monensin sodium
RR: relative recovery
MRT: mean residence time
AUC: area under the concentration–time curve
